# Shared ecological traits influence shape of the skeleton in flatfishes (Pleuronectiformes)

**DOI:** 10.7717/peerj.8919

**Published:** 2020-04-03

**Authors:** Corinthia R. Black, Peter B. Berendzen

**Affiliations:** 1 Department of Biological Sciences, Auburn University, Auburn, AL, USA; 2 Department of Biology, University of Northern Iowa, Cedar Falls, IA, USA

**Keywords:** Body shape, Ecology, Comparative methods, Fish, Marine, Morphological evolution, Geometric morphometrics

## Abstract

In the age of phylogenetic comparative methods, evolutionary biologists have been able to explore evolutionary trends in form in unique and extraordinarily diverse groups of animals. Pleuronectiformes, commonly known as flatfishes, is a diverse and specialized order of fishes that have remarkable asymmetry induced by ocular migration and a benthic life style. Although flatfishes are unique from other fishes, species within the group are morphologically diverse. The origin of ocular migration has been a primary focus of research; however, little is known about overall shape diversification among the flatfishes. In this study, we use integrative methods to examine how body shape evolved within the flatfishes. Shape was quantified from X-rays using geometric morphometrics for 389 individuals across 145 species. The most recent and robust phylogeny was overlaid onto the morphospace and phylogenetic signal was calculated to ascertain convergence in the morphospace. In addition, phylogenetic linear models were employed to determine if ecological traits were correlated with shape and if size had an effect on overall body shape. Results revealed that the majority of variation evolved recently, within the past 15–10-million-years in the middle Miocene, and is highly variable within the flatfishes. These changes are best summarized by body depth, jaw length and medial fin length. Dorsal and anal fin length are correlated, which may be due to the unique mode of locomotion used by flatfishes. A phylogenetic linear model and phylomorphospace analysis suggested that several ecological traits are correlated with shape, which indicates an ecological role in the diversification of flatfishes.

## Introduction

Since the publication of Darwin’s On the Origin of Species, biologists have sought to identify the evolutionary forces driving form. Form can be influenced by many factors, such as ecological interactions, biomechanical constraints and natural selection (Wake & Larson, 1987; Gould, 2002; Adams & Nistri, 2010). The advent of phylogenetic comparative methods provided a mechanistic means to make hypotheses on how different factors influence the evolution of form (Harmon, 2019). In the past two decades, advancements in phylogenetic comparative methods have enabled evolutionary biologists to explore evolutionary trends in form in unique and extraordinarily diverse groups of animals (Adams & Collyer, 2019).

One group of diverse and unique animals is the flatfishes, Pleuronectiformes. Flatfishes are comprised of over 800 species, yet little is understood about the evolution of form within the group. These animals have remarkable asymmetry and lack of intermediate forms, leading some to call them hopeful monsters (Goldshmidt, 1933). Although the origin of sidedness has been extensively studied, little remains understood about how the group diversified following sidedness (Friedman, 2008; Harrington et al., 2016).

Flatfishes comprise a highly specialized order of fishes that displays obvious asymmetry associated with ocular migration and a benthic lifestyle. After hatching bilaterally symmetrical, one eye migrates over the head and rests adjacent to the opposite eye resulting in a laterally flattened body with an eyed side and a blind side. The eyed side is generally pigmented and faces away from the seafloor where the fish resides. Flatfishes are negatively buoyant and spend the majority of their time on the ocean bottom, often buried in the sediment to avoid predators and hide from prey. They have protrusible eyes, which allow flatfishes to see above the substrate where they lie in wait for prey.

**Figure 1:**
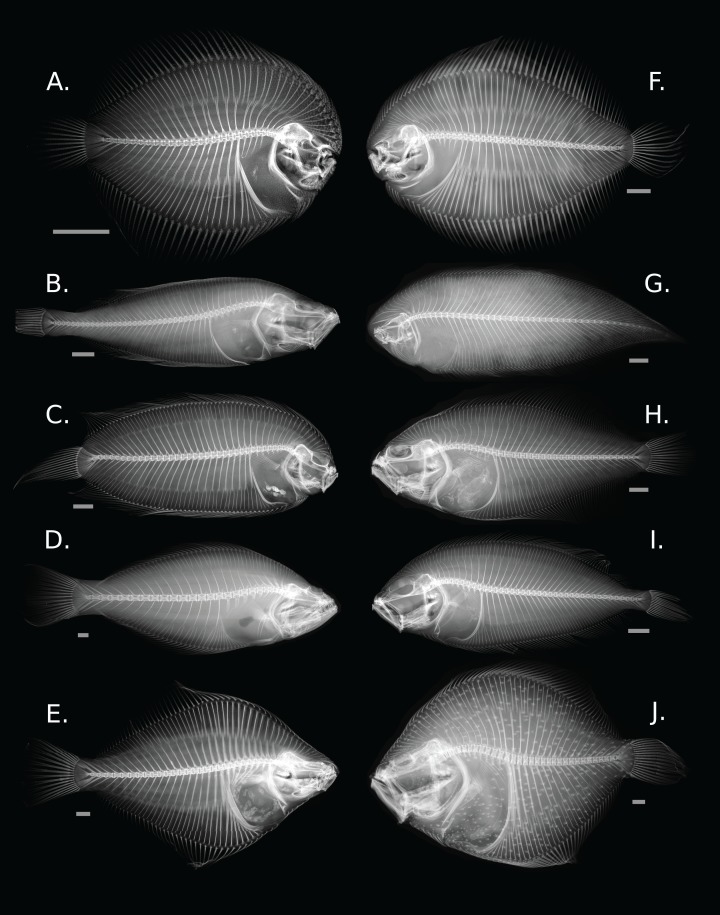
Diversity of skeletal morphology in the flatfishes. (A) *Achirus lineatus* (Achiridae); (B) *Lyopsetta exilis* (Pleuronectidae); (C) *Plagiopsetta glossa* (Samaridae); (D) *Psettodes belcheri* (Psettodidae); (E) *Rhombosolea plebeia* (Pleuronectidae); (F) *Gymnachirus melas* (Achiridae); (G) *Symphurus plagiusa* (Cynoglossidae); (H) *Syacium micrurum* (Paralichthyidae); (I) *Lepidorhombus boscii* (Scophthalmidae); and (J) *Scophthalmus maximus* (Scophthalmidae). Gray bars represent a 1 cm scale.

Flatfishes share several anatomical synapomorphies associated with ocular migration which include cranial asymmetry, an advanced position of the dorsal fin over the cranium, and the presence of a recessus orbitalis, the organ that allows the eyes to extend above the surface of the body (Fig. 1) (Chapleau, 1993; Munroe, 2015). There is a large degree of morphological variation across the order with body shape ranging from fusiform to disk-like (Hensley & Ahlstrom, 1984; Chapleau, 1993; Munroe, 2015). Many species have specialized traits, including a reduction or loss of paired fins, the confluence of medial fins, and the asymmetry of dentition (Gibson et al., 2007).

Despite the wide variation in morphology, flatfishes were historically grouped by direction of sidedness. When the eyes migrate, they come to rest on either the right side (dextral) or the left side (sinistral) of the head (Jordan & Evermann, 1898; Kyle, 1900; Regan, 1910; Norman, 1934; Hubbs, 1945). Most species are either dextral or sinistral. Species with both dextral and sinistral individuals are rare and examples include *Platichthys stellatus*, the starry flounder, and some members of *Psettodes*, the spiny turbots (Chapleau & Amaoka, 1998; Bergstrom & Palmer, 2007). Phylogenetic hypotheses based on morphological and molecular evidence suggested that the traditional classification of the Pleuronectiformes was inaccurate and sidedness alone is an insufficient indicator of relatedness (Hensley & Ahlstrom, 1984; Lauder & Liem, 1985; Chapleau, 1993; Berendzen & Dimmick, 2002; Pardo et al., 2005; Campbell, Chen & López, 2013). Recent molecular phylogenetic hypotheses based on large datasets revealed more complexity to the history of the group, suggesting considerable convergence in morphological traits and sidedness (Friedman, 2008; Palmer, 2009; Betancur-R & Ortí, 2014; Harrington et al., 2016; Byrne, Chapleau & Aris-Brosou, 2018). These comprehensive and robust phylogenies allow for the rigorous study of the evolutionary history of body shape within the group.

Flatfishes reside in all oceans, ranging from the Arctic to Southern oceans with some species that enter brackish water estuaries and others that are found exclusively in fresh water habitats (Kottelat, 1998; Gibson et al., 2007; Duplain, Chapleau & Munroe, 2012; Nelson, Grande & Wilson, 2016). Likewise, their preferred habitat type is widely variable (Gibson et al., 2007; Eschmeyer & Fricke, 2011). Most species can be found at a depth between 0 and 500 m, but some have been recorded at depths below 1,500 m. Flatfishes prefer a variety of benthic habitats, from the mouths of rivers to beyond the continental shelf. Similarly, preferred sediment type and diets are variable among species, with flatfishes burying themselves in a range of substrates, from mud to rocky sediment and diet preferences ranging from polychaetes to other fishes.

The tremendous amount of shape variation and their distinctive asymmetry make flatfishes a unique and interesting group for the analyses of body shape. The processes driving morphological change across the flatfishes are not well understood, and studies examining shape across the phylogeny have yet to be assessed in the group. Previous studies have shown a correlation between shape, phylogeny and ecology. For example, research using cichlid models have examined morphological variation in relation to ecology under an adaptive radiation model. These studies suggested that as ecological niches become available, rapid diversification and convergence in body shape arise (Schluter, 2000; Barluenga et al., 2006; Yoder et al., 2010; Muschick, Indermaur & Salzburger, 2012; Wagner, Harmon & Seehausen, 2012; Elmer et al., 2014; Burress, 2015; Ford et al., 2016; Burress et al., 2018). Phylogenetic hypotheses of flatfishes based on molecular data revealed that flatfishes likely arose through adaptive radiation, yet it is not known if shape is influenced by ecology or other factors (Harrington et al., 2016; Byrne, Chapleau & Aris-Brosou, 2018). By examining shape diversification in the context of genomic hypotheses, we can to observe evolutionary trends in body shape.

The objective of this study was to understand when shape diversification occurred and how ecological traits contributed to shape variation within flatfishes. By incorporating morphological methods in a time-calibrated phylogenetic context, we were able to address how shape diversified across the phylogeny and determine if shape was correlated to diet, the maximum depth zone, climate type, water type and/or sediment type. Using the most robust and recent time-calibrated genomic phylogeny (Byrne, Chapleau & Aris-Brosou, 2018), we generated a phylomorphospace and chronophylomorphospace to visualize shape diversification across the evolutionary history of a representative group of flatfishes (Bookstein, Chernoff & Elder, 1985; Rohlf, 2002; Stone, 2003; Zelditch et al., 2004; Clabaut et al., 2007; Brusatte et al., 2008; Sidlauskas, 2008; Sakamoto & Ruta, 2012). The phylomorphospace allowed us to observe where shape was conserved within a clade and where shape converged. Furthermore, a phylogenetic linear model was employed to determine whether ecological traits correlate to body shape.

## Materials and Methods

### Morphometric analyses

A total of 389 individuals representing 145 species within the Pleuronectiformes were radiographed from fish collections at the University of Kansas Natural History Museum and Smithsonian National Museum of Natural History. Twelve families were represented by the following number of species: Achiridae *n* = 10, Achiropsettidae *n* = 1, Bothidae *n* = 23, Citharidae *n* = 4, Cynoglossidae *n* = 7, Paralichthyidae *n* = 23, Pleuronectidae *n* = 32, Poecilopsettidae *n* = 3, Psettodidae *n* = 2, Samaridae *n* = 2, Scophthalmidae *n* = 3 and Soleidae *n* = 24 (Table S1). When possible, representatives with minimal visible damage and the most recent collection dates were chosen to reduce chances of bone degradation. Between one and six adult individuals for each species were radiographed. The sex of specimens is unknown; however, sexual dimorphism is generally correlated to larger body sizes in females and rarely in variation of shape in flatfishes (Gibson et al., 2007). Additionally, shape variation associated with sex was overpowered by the high diversity in shape across a wide range of species. Radiographs were taken using a Thermo Scientific Kevex PXS5-927EA Microfocus X-ray source, with a focal spot of 4 μm at 2 W, on a Varian PaxScan 4030 E with Kodak Lanex Fine Screen scintillator digital panel with a 40 × 28 cm dimension for Smithsonian specimens. Radiographs of specimens at the University of Kansas were taken using a GE Picker X-ray head in a Technology for Industry controller on Kodax Mammography X-ray film. Images were captured using VIVA K.03 Image Acquisition/Control Software. To reduce distortion of the body caused during the preservation process, specimens were flattened using a sheet of acrylic glass and fabric hook-and-loop fastener straps. Images were manipulated in Photoshop to improve clarity of radiographs by adjusting brightness and color levels.

The diversity in cranial morphology within flatfishes made it difficult to determine homologous landmarks across the species included in this study. However, we were able to identify ten landmarks defining the body outline (Fig. S1). These landmarks were chosen based on standard landmarks used in geometric morphometric studies of fishes and the ability to capture the overall outline of the flatfish (Zelditch et al., 2004). Flatfishes also have a curvature of the spine which primarily involves the abdominal vertebrae. As the number of vertebrae change across species, the curvature was captured using a series of landmarks and semi-landmarks. The semi-landmarks were evenly spaced between the vertebral landmarks along the length of the spinal column. Semi-landmarks are not individually homologous, instead they sample points along the homologous curve of the spinal column (Zelditch et al., 2004). Landmarks and the curve were digitized using the software TPSdig 2.16 (Rohlf, 2010) and semi-landmarks were appended to landmark files using tpsUtil (Rohlf, 2010).

Specimens were superimposed using a generalized least squares Procrustes superimposition to remove non-shape related information (translation, orientation and size) using geomorph 3.0.5 in R (Adams & Otárola-Castillo, 2013). The superimposed landmarks were then averaged in the base package in R (R Development Core Team, 2016) for each species.

### Morphospace methods

A principal component analysis (PCA) was performed for averaged data in the R package geomorph and principal component backtransformations were generated (Olsen, 2017) to view theoretical shape of the morphospace for 145 species. To explore evolutionary trends of body shape within the flatfishes, a phylomorphospace was generated in the R package geomorph (Adams & Otárola-Castillo, 2013). This method projects the phylogeny onto the multivariate morphospace so the magnitude and direction of shape change can be interpreted in a phylogenetic context (Sidlauskas, 2008). The phylogenomic tree (Fig. S2) (Byrne, Chapleau & Aris-Brosou, 2018) was downloaded from GitHub (https://github.com/sarisbro) and input into the R environment. Non-corresponding specimens were pruned from the tree in the R package ape (Paradis, Claude & Strimmer, 2004), leaving 98 corresponding species in 15 identifiable clades. Where necessary, clades that separated known families were labeled as 1 and 2 (Paralichthyidae 1 and Paralichthyidae 2). The phylomorphospace was generated by overlaying the pruned tree onto the PCA. To visually understand temporal changes in shape across the phylogeny, time was added to the *z*-axis to generate a chronophylomorphospace. This method plots reconstructed ancestral shapes in the morphospace and across time based on known relationships (Sakamoto & Ruta, 2012).

### Phylogenetic signal, allometry and phylogenetic linear model

Phylogenetic signal was calculated using the K_mult_ method in geomorph (Adams & Otárola-Castillo, 2013). The K_mult_ method is a mathematical generalization of the Kappa statistic (Blomberg, Garland & Ives, 2003), and uses a Brownian motion model to evaluate the degree of phylogenetic signal in a dataset (Adams & Otárola-Castillo, 2013). This is the most appropriate method to use for multivariate data (Adams, 2014; Adams & Collyer, 2018a).

An allometric regression was performed to estimate the effect of centroid size and shape using a simple allometric linear model (shape coordinates ~ log(size)) and a unique family allometric model (shape coordinates ~ log(size) × family) with procD.lm in geomorph (Adams & Collyer, 2018a). To test the amount of shape variation affected by size, we calculated the morphological disparity for shape with and without size correction (shape coordinates ~ log(size) × family vs. shape coordinates ~ family) in geomorph and preformed a simple linear regression to determine if size significantly affected shape. Additionally, we obtained fin lengths using interlmkdist in geomorph and performed a simple regression to determine if dorsal and anal fin lengths correlated to one another.

Primary ecological data (diet, maximum depth zone, climate type, water type and preferred sediment type) were compiled from FishBase (Froese & Pauly, 2019) and a phylogenetic linear model was performed in geomorph using the procD.pgls and pairwise functions (Adams & Collyer, 2018b). When FishBase suggested multiple ecological variables, the primary variable(s) was used. In the case that FishBase suggested two or more variables were equally primary, the ecological variables were considered independent from other variables to account for the unique ecological rank. For example, if diet type was 40% fish, 40% crustaceans and 20% polychaetes, the assigned diet type would be “fish and crustaceans”. Where possible, ecological traits in question were cross referenced to the literature, and species without reliable traits were pruned from the dataset.

Phylogenetic linear models calculate the amount of shape variation and the estimated probability of variation attributed to ecological factors in a linear model to detect relationships between shape and ecological traits. A linear model comparing shape to ecological traits was used (shape coordinates ~ depth + climate + water type + diet + sediment) and type III (marginal) sums of squares (SS) was calculated. Type III SS was computed as the effect of each variable was evaluated after other factors, which means the order of factors in the linear model does not affect the outcome. However, this method is not appropriate for missing data, so we removed taxa which were missing ecological data. A pairwise test was implemented to identify which ecological types were different from one another. For all tests, a randomized residual permutation procedure with 1,000 permutations was used. Ecological traits that significantly correlated to shape were plotted to the phylomorphospace to visualize trends in shape and ecology.

## Results

### Morphospace

**Figure 2:**
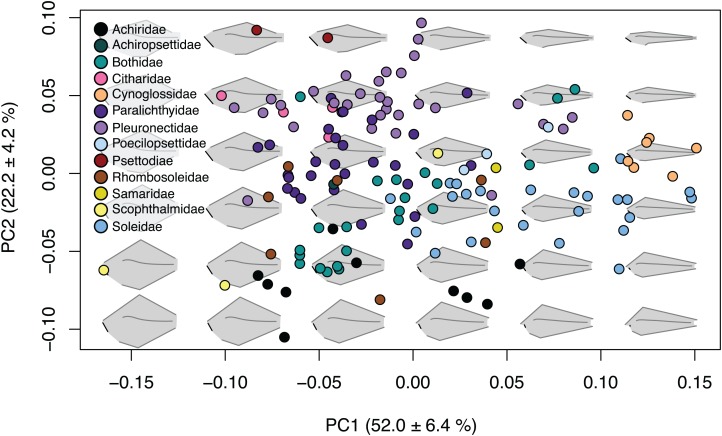
Body shape variation within flatfishes. The morphospace biplot of PCs 1 and 2 represents 74.2% of the body shape variation within the flatfishes. Each point indicates the mean of a species with colors matching the family depicted in the key. Backtransformed shapes (in gray) portray shape variation throughout morphospace with fin length, jaw length and spinal curvature represented as black lines on shapes.

The first four principal components (Table S2) account for 85.3% of shape variation and were associated with body depth at the location of the first caudal vertebrae, length of the jaw and origin and insertion points of the caudal and anal fins (Fig. 2; Fig. S3). Principal component one describes 52.0 ± 6.4% of the variation, and principal component two explains 22.2 ± 4.2% of shape variation. Species that fell towards the negative ends of PC1 and PC2 are very round in body shape, whereas species at the positive ends of PC1 and PC2 have oblong body shapes (Fig. 2). Similarly, species that are on the negative end of PC1 and positive end of PC2 have long jaws and short dorsal and anal fins, whereas species on the positive end of PC1 and negative end of PC2 have short jaws and long dorsal and anal fins (Fig. 2). Principal component three describes 6.4 ± 2.2% of shape variation and principal component four describes 4.7 ± 1.9% of shape variation. Shape change across PC3 and PC4 is similar to PC1 and PC2, yet less extreme. Species that fell to the negative end of PC3 have long jaws and short dorsal and anal fins whereas the positive end shows species with short jaws and short dorsal and anal fins. PC4 shows species which fell to the negative end have long jaws with oblong bodies, and species that fell to the positive end have short jaws with deep bodies (Fig. S3).

### Phylomorphospace

**Figure 3:**
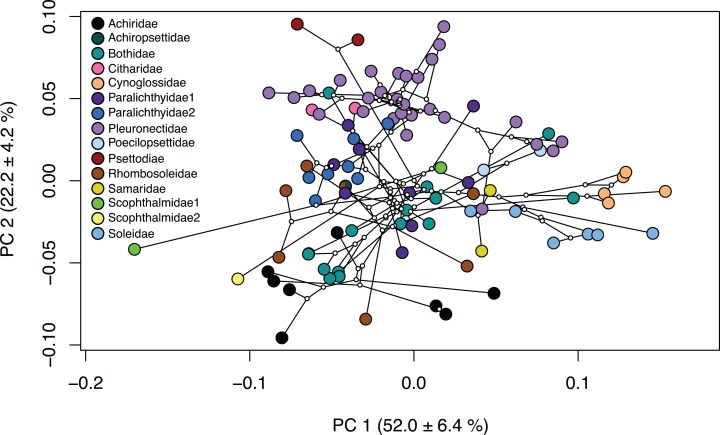
Phylomorphospace of body shape within flatfishes. The genomic phylogeny (Byrne, Chapleau & Aris-Brosou, 2018) projected onto the morphospace to demonstrate the evolutionary relationships of body shape variation within the flatfishes. Solid colored points indicate the mean of a species with ancestral nodes represented by small white circles.

Several families within the pleuronectiforms were clustered together in the phylomorphospace; however, there is overlap within space for most families. Psettodidae (maroon), Citharidae (pink) and Poecilopsettidae (light blue), Samaridae (gold), Cynoglossidae (orange) and Soleidae (medium blue) show clustering within the family and separation from other families across the PCA (Fig. 3; Fig. S4). Conversely, Achiridae (black), Bothidae (medium green), Paralichthyidae 1 (dark purple), Paralichthyidae 2 (dark blue), Pleuronectidae (light purple), Rhombosoleidae (brown) and Schophthalmidae 1 (lime green) are widespread across the PCA and display body shapes and jaw lengths of all types (Fig. 3; Fig. S4). Furthermore, Bothidae and Rhombosoleidae are widespread across PC3 and PC4, whereas other families cluster closely (Fig. S5). Families with shapes on the extreme ends include Cynoglossidae and Soleidae which cluster to the far positive end of PC1, sharing oblong bodies with small jaws and long fins. Psettodidae has an oblong body with a long jaw on the far positive end of PC2, whereas Achiridae has a round body with a short jaw toward the negative end of PC2 (Fig. 3; Fig. S4). Clustering of families is more clearly shown in a three-dimensional PCA (Fig. S6).

### Chronophylomorphospace

**Figure 4:**
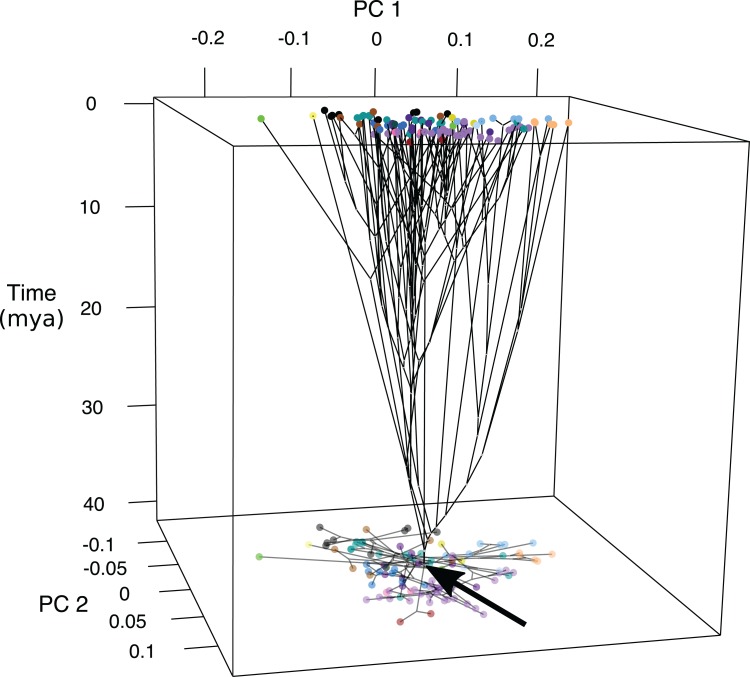
Chronophylomorphospace of body shape within flatfishes. The time-calibrated genomic phylogeny (Byrne, Chapleau & Aris-Brosou, 2018) mapped onto the morphospace with the time in millions of years depicted on the *z*-axis. Colored points indicate the mean of a species and the black arrow indicates the root of the phylogeny. A two-dimensional morphospace is represented as a shadow at the bottom of the graph.

Ancestral states were inferred at the nodes of the genomic tree (Fig. S2) (Byrne, Chapleau & Aris-Brosou, 2018) and time was plotted as axis *z* (Fig. 4; Fig. S7). Early divergence led to changes in fin length and jaw shape between approximately 40 and 30 MYA with the lineage leading to Psettodiae becoming slightly more oblong with shorter fins and longer jaws, and the lineages leading to Cynoglossidae and Soleidae becoming oblong with longer fins and shorter jaws. At approximately 30 MYA body shape changed across the PC2 axis with changes in jaw length and body depth. The majority of shape diversification occurred 15–10 MYA, where species experienced changes across PC1 in addition to PC2 to become widespread across the phylomorphospace (Fig. 4; Fig. S7).

### Phylogenetic signal allometry and phylogenetic linear model

**Figure 5:**
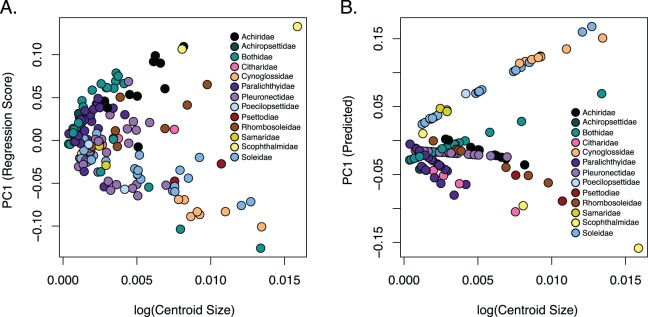
Relationship of body shape and size in flatfishes. (A) Scatterplot of the regression of body shape on the log centroid size and (B) the predicted shape values from regression scores for each family. Each dot indicates the mean of a species with colors coordinating to the family depicted in the key.

The observed phylogenetic signal was lower than the expected signal (1.0) under a Brownian motion model at 0.6161 and was significant with a *p*-value of 0.001. A comparison of allometric models showed that unique family allometries (*p* = 0.001) are appropriate. Larger species in the families Achiridae, Citharidae, Paralichthyidae, Pleuronectidae, Psettodiae, Rhombosoleidae, Samaridae and Scophthalmidae have deeper bodies, longer jaws and short dorsal and anal fins, whereas larger species in the families Bothidae, Cynoglossidae, Poecilopsettidae and Soleidae have oblong bodies with shorter jaws and long dorsal and anal fins (Fig. 5). A linear regression of morphological disparity with and without size correction showed that size did not significantly affect the Procrustes variance (*p* = 0.08) which suggests shape should not be corrected for allometry. Additionally, a simple regression showed that the dorsal and anal fin lengths were significantly correlated (*p* < 2E−16); as the dorsal fin increases in length the anal fin increases (Fig. S8).

A phylogenetic linear model was used to determine the relationship between ecology and shape while accounting for phylogeny. Ecological factors that were significant included water type (*p* = 0.003), diet (*p* = 0.003), and sediment type (*p* = 0.023) (Table S3). Pairwise tests determined the body shape of flatfishes which are found on mud sediment types were significantly different (*p* = 0.003) from flatfishes which are generalists (found on mud, sand, or rock) and the body shapes of flatfishes which preferred sand sediments were also significantly different (*p* = 0.005) from generalists which can be found on mud, sand and rock sediment types (Table S4). Although water type and diet were significant in the phylogenetic linear model, pairwise tests showed no significant differences between groups (Table S4). Significant ecological traits did not visually cluster across the phylomorphospace (Fig. S9).

## Discussion

This study is the first to use a well-supported and robust genomic phylogeny to analyze skeletal body shape in a phylogenetic context of the Pleuronectiforms. Geometric morphometric analyses captured body shape diversification across the order and incorporated phylogenetic hypotheses to reveal that evolutionary history and ecological traits are important for body variation. Shape is highly variable within the flatfishes, and is best summarized by changes in body depth, jaw length and medial fin length. Dorsal and anal fin length are correlated, either both elongated or shortened across the morphospace, which may be associated to the unique mode of locomotion in flatfishes. A phylogenetic linear model showed that shape is correlated to ecological traits which may suggest ecology is driving shape. Finally, the majority of variation evolved recently within the past 15–10-million-years in the middle Miocene.

Flatfishes are morphologically diverse and range in shape from oblong to rounded with short to long jaws and short to long dorsal and anal fins. Although flatfishes can have any combination of body depth and jaw length, fin length is restricted; the dorsal fin is always longer than the anal fin, and as the dorsal fin increases in length, the anal fin always increases in length (Fig. S8). A linear model shows that dorsal and anal fin length are correlated which may be related to their unique mode of locomotion. Flatfishes use a *Tetraodontiform* mode of locomotion, the simultaneous use of dorsal and anal fins, in swimming, walking and burial behaviors (Sfakiotakis, Lane & Davies, 1999; Munroe, 2015; Fox et al., 2018). Fin length may be restricted to optimize movement across the sediment which may not be as effective if the dorsal fin was shorter than the anal fin. However, this remains to be tested and awaits further research.

Flatfishes also range in length from 4.5 cm (*Tarphops oligolepis*) to 2.5 m (*Hippoglossus hippoglossus*) (Chapleau & Amaoka, 1998). An allometric regression showed that size affects shape although the amount of variation in shape that can be attributed to size is very small. Furthermore, our dataset is biased toward smaller specimens (less than 45 cm in length) as we were restricted the size of the X-ray machine. Interestingly, larger species in the families Achiridae, Citharidae, Paralichthyidae, Pleuronectidae, Psettodiae, Rhombosoleidae, Samaridae and Scophthalmidae have deeper bodies, longer jaws and short dorsal and anal fins, whereas larger species in the families Bothidae, Cynoglossidae, Poecilopsettidae and Soleidae have oblong bodies with shorter jaws and long dorsal and anal fins (Fig. 5). Although more data is needed to clarify patterns within families, we were able to demonstrate that size/shape patterns vary across families within the flatfishes and that the effect of size on shape was negligible.

Furthermore, shape and ecological traits are correlated, suggesting that ecology may drive shape in the flatfishes. This is supported by a small phylogenetic signal and significant relationships for water type, diet, and sediment type indicated by the phylogenetic linear model. Although the phylogenetic linear model was significant for several ecological traits, pairwise tests showed no significate differences between groups with the exception for sediment type. The shape of flatfishes which are found on mud sediment and the shape of flatfishes which are found on sand sediment were significantly different from generalists (prefer all sediments types). This suggests that flatfishes which are sediment specialists are different in shape from flatfishes which are generalists. We are unable to identify specific trends in shape and ecology as there was a lack of clustering by ecological trait in the phylomorphospace (Fig. S9C) and a lack of support for pairwise distances in most ecological types. More robust analyses that focus on ecological traits in flatfishes are needed to address how shape is correlated to ecological traits among species.

Chronophylomorphospace results suggest that the majority of shape variation evolved within the past 15–10-million-years during the middle Miocene (Fig. 4; Fig. S7). Early divergence led to changes in fin length and jaw shape between approximately 40 and 30 MYA for lineages leading to the Psettodiae and the clade containing Cynoglossidae and Soleidae. The Psettodiae became slightly more oblong with shorter fins and longer jaws, and the ancestor of Cynoglossidae and Soleidae became oblong with longer fins and shorter jaws. The middle Miocene marks a time of decreasing temperatures and is often referred to as the middle Miocene disruption. During this time there was a wave of aquatic extinctions which may have led to a speciation and diversification event in the flatfishes.

## Conclusions

The Pleuronectiformes is a highly diverse order, with variation in shape best summarized by changes in body depth, jaw length and medial fin length and is likely influence by ecological traits. Further research into how dorsal and anal fin length influence locomotion is needed to determine if and why dorsal and anal fin lengths are correlated. Overall, the Pleuronectiformes are incredibly diverse in both shape and ecological traits resulting in a vast geographical and ecological distribution.

## Supplemental Information

10.7717/peerj.8919/supp-1 Supplemental Information 1 List of specimens used in this study. A complete list of specimen accessions and number of specimens included in study. All specimens are accessioned in fish collections at the University of Kansas Natural History Museum and Smithsonian National Museum of Natural History. Bolded specimens are included in the phylomorphospace and chronophylomorphospace.

10.7717/peerj.8919/supp-2 Supplemental Information 2 Summary of principal components as shown by the principal component analysis. The proportion of variance and standard deviation are listed for the first 31 principal components.

10.7717/peerj.8919/supp-3 Supplemental Information 3 Correlation of ecological traits to shape as indicated by Procrustes ANOVA. Significant ecological factors are highlighted in green. Significants codes as follows (***) 0; (**) 0.001; (*) 0.01; (.) 0.05.

10.7717/peerj.8919/supp-4 Supplemental Information 4 Pairwise distances between ecological groups in the context of shape. Ecological group comparisons that are significant are highlighted in green. Significants codes as follows (***) 0; (**) 0.001; (*) 0.01; (.) 0.05.

10.7717/peerj.8919/supp-5 Supplemental Information 5 Landmarks used in this study. (1) anterior tip of premaxilla; (2) quadrate and articular junction; (3) origin of anterior dorsal fin; (4) dorsal basal bone between interneural spines of the first caudal vertebrae; (5) insertion of dorsal fin; (6) origin of caudal fin; (7) insertion of caudal fin; (8) insertion of anal fin; (9) ventral basal bone between interneural spines of the first caudal vertebrae; (10) origin of anal fin; (11) first abdominal vertebrae midpoint; (12) first caudal vertebrae midpoint; (13) urostyle midpoint. Curve containing 25 semi-landmarks following the spinal column. *Syacium micrurum (*Paralichthyidae).

10.7717/peerj.8919/supp-6 Supplemental Information 6 Genomic phylogeny (Byrne, Chapleau & Aris-Brosou, 2018) pruned to match geometric morphometric dataset. Colors correlate to distinct clades in morphospace, phylomorphospace, and chronophylomorphospace.

10.7717/peerj.8919/supp-7 Supplemental Information 7 Body shape variation of the skeletal shape of 145 species within the flatfishes revealed by principal components analysis (PCA) for PCs 3 and 4. The morphospace biplot of PCs 3 and 4 represents the overall body shape variation within the flatfishes. Backtransform shapes (gray) portray shape variation throughout morphospace and fin length, jaw length and spinal curvature are shown on backtransform shapes as black lines.

10.7717/peerj.8919/supp-8 Supplemental Information 8 Interactive phylomorphospace based on a genomic phylogeny (Byrne, Chapleau & Aris-Brosou, 2018). Phylomorphospace of the genomic phylogeny (Byrne, Chapleau & Aris-Brosou, 2018) for 98 species of flatfishes. Click on the colored circle next to the family name to circle the group. Show name of species by hovering over points on PCA.

10.7717/peerj.8919/supp-9 Supplemental Information 9 Phylomorphospace of PCs 3 and 4 for 98 species of flatfishes. The genomic phylogeny (Byrne, Chapleau & Aris-Brosou, 2018) was mapped onto the morphospace biplot of PCs 1 and 2. Colors correlate to distinct clades.

10.7717/peerj.8919/supp-10 Supplemental Information 10 Interactive three dimensional phylomorphospace of the genomic phylogeny (Byrne, Chapleau & Aris-Brosou, 2018) for 98 species of flatfishes. The genomic phylogeny was mapped onto the three dimensional morphospace for PCs 1, 2 and 3. Colors correlate to distinct clades.

10.7717/peerj.8919/supp-11 Supplemental Information 11 Interactive three dimensional chronophylomorphospace based on a genomic phylogeny (Byrne, Chapleau & Aris-Brosou, 2018) for 98 species of flatfishes. The genomic phylogeny was mapped onto the morphospace with the *z*-axis depicting time in millions of years. The two-dimensional morphospace is represented as a shadow at the bottom.

10.7717/peerj.8919/supp-12 Supplemental Information 12 Observed *k* value of phylogenetic signal. Phylogenetic signal is shown on *x*-axis and frequency on *y*-axis. Observed *k* value is noted along the *x*-axis by black arrow.

10.7717/peerj.8919/supp-13 Supplemental Information 13 Ecological traits mapped to phylomorphospace. (A) climate type, (B) diet type, (C) water type and (D) sediment type. Colors correlate to distinct clades.

## Additional Information and Declarations

### Data Availability

The following information was supplied regarding data availability: Raw data, code, and Supplemental Files are available at GitHub: https://github.com/corinthiablack/Black-and-Berendzen_Flatfish-Shape. A complete list of specimen accessions is available in Table S1. All specimens are accessioned in fish collections at the University of Kansas Natural History Museum and Smithsonian National Museum of Natural History.
