# Supplementary material for: Shared ecological traits influence shape of the skeleton in flatfishes (Pleuronectiformes)
Source: PeerJ. 2020 Apr 3;8:e8919. doi: 10.7717/peerj.8919 (PMC7134016; doi:10.7717/peerj.8919)
Supplement: Supplemental Information 1 — A complete list of specimen accessions and number of specimens included in study. All specimens are accessioned in fish collections at the University of Kansas Natural History Museum and Smithsonian National Museum of Natural History. Bolded specimens are included in the phylomorphospace and chronophylomorphospace. [file peerj-08-8919-s001.docx]

| **Taxon** | **Catalog Number (Count)** |
| --- | --- |
| Pleuronectiformes |  |
| Achiridae |  |
| ***Achirus declivis*** | **USNM 286840 (2)** |
| ***Achirus lineatus*** | **USNM 156403 (2)** |
| *Achirus mazatlanus* | KU 22694 (2) |
| ***Apionichthys dumerili*** | **USNM 233556 (1), USNM 233588 (1)** |
| ***Catathuridium jenunsii*** | **USNM 55583 (2), USNM 181499 (1)** |
| ***Gymnachirus melas*** | **KU 30098 (1), KU 30120 (1), USNM 291088 (3)** |
| ***Gymnachirus texae*** | **KU 29675 (1), KU 29675 (1), USNM 158296 (1) USNM 358229 (1)** |
| ***Hypoclinemus mentalis*** | **USNM 167720 (1), USNM 191555 (2)** |
| *Nodogymnus fasciatus* | USNM 152033 (1) |
| ***Trinectes maculatus*** | **USNM 15091 (1), USNM 34837 (2)** |
| Achiropsettidae |  |
| ***Mancopsetta maculata*** | **USNM 362523 (1), USNM 362528 (2)** |
| Bothidae |  |
| ***Arnoglossus imperialis*** | **USNM 282031 (3), USNM 357926 (3)** |
| ***Arnoglossus laterna*** | **USNM 282245 (3)** |
| *Asterorhombus cocosensis* | USNM 260366 (3), USNM 362478 (1) |
| ***Bothus lunatus*** | **USNM 282590 (1), USNM 349048 (1), USNM 359466 (1)** |
| ***Bothus myriaster*** | **USNM 375617 (3)** |
| ***Bothus podas*** | **KU 19935 (1)** |
| ***Bothus robinsi*** | **USNM 159614 (3)** |
| ***Chascanopsetta lugubris*** | **USNM 282744 (1)** |
| ***Crossorhombus azureus*** | **USNM 260395 (3)** |
| *Engyophrys sanctilaurentii* | USNM 375570 (3) |
| ***Engyprosopon grandisquama*** | **USNM 56384 (3)** |
| ***Grammatobothus polyophthalmus*** | **USNM 260448 (1), USNM 260449 (1), USNM 260481 (1)** |
| ***Laeops kitaharae*** | **USNM 362498 (1)** |
| ***Laeops nigromaculatus*** | **USNM 307566 (3)** |
| *Monolene atrimana* | USNM 159442 (3) |
| ***Parabothus chlorospilus*** | **USNM 394618 (3)** |
| *Pelecanichthys crumenalis* | USNM 55256 (3) |
| *Perissias taeniopterus* | USNM 362514 (1), USNM 362515 (1) |
| *Platophrys* | USNM 169911 (2) |
| ***Psettina gigantea*** | **USNM 260446 (1), USNM 260482 (1)** |
| *Taeniopsetta radula* | USNM 394619 (3) |
| *Trichopsetta caribbaea* | USNM 159579 (3) |
| ***Trichopsetta ventralis*** | **USNM 159510 (1), USNM 395224 (2)** |
| Citharidae |  |
| *Brachypleura novaezeelandiae* | USNM 261526 (3) |
| ***Citharoides macrolepis*** | **KU 27264 (2), USNM 308017 (3)** |
| ***Citharus linguatula*** | **USNM 362482 (1), USNM 362485 (2), USNM 397277 (3)** |
| *Lepidoblepharon ophthalmolepis* | USNM 127409 (1) |
| Cynoglossidae |  |
| *Arelia bilineata* | USNM 203758 (3) |
| ***Cynoglossus arel*** | **USNM 203995 (1)** |
| ***Cynoglossus interruptus*** | **KU 27260 (1)** |
| ***Symphurus atricaudus*** | **USNM 38018 (3)** |
| *Symphurus bathyspilus* | USNM 138062 (3) |
| ***Symphurus civitatium*** | **USNM 157694 (2), USNM 158278 (1)** |
| ***Symphurus plagiusa*** | **USNM 316767 (3)** |
| Paralichthyidae |  |
| *Ancylopsetta cycloidea* | USNM 282409 (1), USNM 282411 (1), USNM 282412 (1) |
| *Ancylopsetta dilecta* | KU 30118 (1) |
| *Ancylopsetta ommata* | USNM 93598 (2), USNM 125387 (1), USNM 156077 (1) |
| *Azevia panamensis* | USNM 81038 (1) |
| ***Citharichthys arctifrons*** | **USNM 29064 (1)** |
| ***Citharichthys gilberti*** | **KU 40338 (2)** |
| ***Citharichthys macrops*** | **KU 5112 (1)** |
| ***Citharichthys sordidus*** | **USNM 77968 (3)** |
| ***Citharichthys stigmaeus*** | **KU 23709 (1)** |
| ***Cyclopsetta chittendeni*** | **USNM 155724 (1), USNM 156026 (1), USNM 156028 (1)** |
| ***Etropus crossotus*** | **USNM 93611 (1), USNM 300513 (2)** |
| ***Etropus microstomus*** | **USNM 119050 (3)** |
| *Gastropsetta frontalis* | USNM 286092 (1), USNM 286096 (2) |
| *Hippoglossina bollmani* | USNM 362262 (1), USNM 362276 (2) |
| *Lioglossina tetrophthalmus* | USNM 362503 (1), USNM 375893 (1) |
| ***Paralichthys adspersus*** | **USNM 362302 (3)** |
| ***Paralichthys albigutta*** | **USNM 157642 (3)** |
| ***Paralichthys californicus*** | **USNM 54775 (3)** |
| *Paralichthys lethostigma* | KU 20072 (1) |
| ***Pseudorhombus arsius*** | **USNM 375500 (3)** |
| ***Pseudorhombus pentophthalmus*** | **USNM 71465 (3)** |
| *Syacium gunteri* | USNM 118643 (3) |
| ***Syacium micrurum*** | **USNM 286626 (3)** |
| ***Tarphops oligolepis*** | **KU 27269 (1), USNM 77071 (1), USNM 152478 (2)** |
| ***Tephrinectes sinensis*** | **USNM 86372 (3), USNM 87056 (1)** |
| *Thysanopsetta naresi* | USNM 77392 (1), USNM 103793 (1) |
| ***Xystreurys liolepis*** | **USNM 41906 (1), USNM 46317 (1)** |
| Pleuronectidae |  |
| *Acanthopsetta nadeshnyi* | USNM 77114 (1), USNM 77118 (1), USNM 77123 (1) |
| ***Atheresthes stomias*** | **USNM 125529 (1)** |
| ***Cleisthenes herzensteini*** | **USNM 77093 (1), USNM 77095 (1), USNM 77097 (1)** |
| ***Cleisthenes pinetorum*** | **USNM 77089 (1), USNM 150375 (3)** |
| ***Drepanopsetta platessoides*** | **USNM 197612 (3)** |
| ***Embassichthys bathybius*** | **USNM 150190 (1), USNM 187656 (1)** |
| ***Eopsetta grigorjewi*** | **USNM 71960 (1), USNM 77081 (1), USNM 77083 (1)** |
| ***Eopsetta jordani*** | **USNM 27499 (1), USNM 46429 (1), USNM 365701 (1)** |
| ***Glyptocephalus cynoglossus*** | **USNM 261360 (2), USNM 261527 (3)** |
| ***Glyptocephalus zachirus*** | **USNM 306352 (3)** |
| ***Hippoglossoides dubius*** | **USNM 77059 (1), USNM 77061 (2)** |
| ***Hippoglossoides elassodon*** | **USNM 60659 (3)** |
| ***Hippoglossus hippoglossus*** | **USNM 39743 (1), USNM 54300 (1), USNM 163652 (1)** |
| *Hypsopsetta guttulata* | USNM 286147 (1) |
| ***Isopsetta isolepis*** | **USNM 54037 (3)** |
| ***Kareius bicoloratus*** | **USNM 56373 (1), USNM 71997 (2)** |
| ***Lepidopsetta bilineata*** | **USNM 76430 (3)** |
| ***Limanda limanda*** | **USNM 261534 (3)** |
| *Liopsetta glacialis* | USNM 29928 (1), USNM 48630 (2) |
| ***Lyopsetta exilis*** | **USNM 60632 (1), USNM 63562 (3)** |
| ***Microstomus pacificus*** | **USNM 46411 (2), USNM 63573 (1)** |
| ***Parophrys vetulus*** | **USNM 46435 (3), USNM 127075 (2)** |
| ***Platichthys flesus*** | **USNM 10031 (3)** |
| ***Platichthys stellatus*** | **USNM 54485 (3)** |
| ***Pleuronectes platessa*** | **USNM 197577 (3)** |
| *Protopsetta herzensteini* | USNM 71961 (1), USNM 71996 (1) |
| ***Psettichthys melanostictus*** | **USNM 36894 (1), USNM 67272 (1)** |
| ***Pseudopleuronectes americanus*** | **USNM 48972 (3)** |
| ***Pseudopleuronectes herzensteini*** | **USNM 77181 (1), USNM 77182 (3)** |
| ***Reinhardtius hippoglossoides*** | **USNM 286576 (1)** |
| ***Tanakius kitaharae*** | **USNM 77162 (1), USNM 77165 (2)** |
| ***Verasper moseri*** | **USNM 49456 (3)** |
| Poecilopsettidae |  |
| *Poecilopsetta albomarginata* | USNM 159446 (3) |
| ***Poecilopsetta beanii*** | **USNM 164146 (3)** |
| ***Poecilopsetta plinthus*** | **USNM 77186 (2), USNM 150688 (1)** |
| Psettodidae |  |
| ***Psettodes belcheri*** | **USNM 286357 (1), USNM 286358 (1), USNM 286359 (1)** |
| ***Psettodes erumei*** | **USNM 36896 (1), USNM 122016 (1), USNM 122017 (1), USNM 345415 (1), USNM 361608 (1)** |
| Rhombosoleidae |  |
| ***Ammotretis rostratus*** | **USNM 282708 (3)** |
| ***Oncopterus darwinii*** | **USNM 86732 (1)** |
| ***Pelotretis flavilatus*** | **USNM 176808 (3)** |
| ***Peltorhamphus novaezeelandiae*** | **USNM 320592 (3)** |
| ***Rhombosolea leporina*** | **USNM 304937 (1)** |
| ***Rhombosolea plebeia*** | **USNM 176810 (3)** |
| ***Rhombosolea tapirina*** | **USNM 286578 (3)** |
| Samaridae |  |
| ***Plagiopsetta glossa*** | **USNM 396096 (1)** |
| ***Samariscus longimanus*** | **USNM 137384 (1), USNM 137385 (2)** |
| Scophthalmidae |  |
| ***Lepidorhombus boscii*** | **USNM 286177 (3)** |
| ***Scophthalmus aquosus*** | **USNM 91255 (3)** |
| ***Scophthalmus maximus*** | **USNM 22996 (1), USNM 25963 (2)** |
| Soleidae |  |
| *Achiroides melanorhynchus* | USNM 230355 (2) |
| *Amate japonica* | USNM 71608 (1), USNM 72090 (1) |
| *Aseraggodes cyaneus* | USNM 137676 (1), USNM 137677 (1) |
| ***Aseraggodes kobensis*** | **USNM 71464 (3), USNM 286826 (1)** |
| *Bathysolea polli* | USNM 286834 (1), USNM 286835 (2) |
| *Brachirus aenea* | USNM 305762 (3) |
| *Brachirus aspilos* | USNM 137679 (1) |
| *Dexillus muelleri* | USNM 22853 (1), USNM 291084 (1), USNM 291085 (1) |
| ***Dicologlossa hexophthalma*** | **USNM 290983 (1), USNM 290985 (2)** |
| *Euryglossa orientalis* | USNM 291012 (1) |
| *Euryglossa sorsogonensis* | USNM 340538 (1) |
| *Liachirus melanospilos* | USNM 76657 (1), USNM 236108 (3) |
| *Microchirus frechkopi* | USNM 274752 (2), USNM 274759 (1) |
| *Monochirus monochir* | USNM 34359 (2) |
| *Parachirus xenicus* | USNM 218768 (3) |
| *Pardachirus balius* | USNM 306429 (2) |
| ***Pegusa impar*** | **USNM 291007 (1), USNM 291006 (1), USNM 291008 (2)** |
| *Phyllichthys sclerolepis* | USNM 174031 (1) |
| ***Pseudaesopia japonica*** | **USNM 56372 (1)** |
| *Soleichthys microcephalus* | USNM 47886 (2), USNM 59956 (1) |
| *Soleidae* | USNM 291140 (2) |
| ***Synapturichthys kleinii*** | **USNM 291009 (1), USNM 291101 (1)** |
| *Vanstraelenia chirophthalma* | USNM 274741 (3) |
| ***Zebrias fasciatus*** | **USNM 191154 (1)** |
