# Supplementary material for: Shared ecological traits influence shape of the skeleton in flatfishes (Pleuronectiformes)
Source: PeerJ. 2020 Apr 3;8:e8919. doi: 10.7717/peerj.8919 (PMC7134016; doi:10.7717/peerj.8919)
Supplement: Supplemental Information 2 — The proportion of variance and standard deviation are listed for the first 31 principal components. [file peerj-08-8919-s002.docx]

| **PC Summary: Importance of first k=31 (out of 76) components** | | | | | |
| --- | --- | --- | --- | --- | --- |
|  | PC1 | PC2 | PC3 | PC4 | PC5 |
| Standard deviation | 0.06396 | 0.04177 | 0.02235 | 0.01917 | 0.01665 |
| Proportion of Variance | 0.52036 | 0.22188 | 0.06355 | 0.04673 | 0.03527 |
| Cumulative Proportion | 0.52036 | 0.74224 | 0.80579 | 0.85252 | 0.88779 |
|  | PC6 | PC7 | PC8 | PC9 | PC10 |
| Standard deviation | 0.01493 | 0.01399 | 0.01088 | 0.009383 | 0.007987 |
| Proportion of Variance | 0.02835 | 0.0249 | 0.01505 | 0.0112 | 0.00811 |
| Cumulative Proportion | 0.91614 | 0.94104 | 0.95609 | 0.96729 | 0.9754 |
|  | PC11 | PC12 | PC13 | PC14 | PC15 |
| Standard deviation | 0.006299 | 0.005869 | 0.005466 | 0.004784 | 0.004076 |
| Proportion of Variance | 0.00505 | 0.00438 | 0.0038 | 0.00291 | 0.00211 |
| Cumulative Proportion | 0.98045 | 0.98483 | 0.98863 | 0.99154 | 0.99365 |
|  | PC16 | PC17 | PC18 | PC19 | PC20 |
| Standard deviation | 0.003446 | 0.003128 | 0.002615 | 0.002341 | 0.002154 |
| Proportion of Variance | 0.00151 | 0.00124 | 0.00087 | 0.0007 | 0.00059 |
| Cumulative Proportion | 0.99516 | 0.99641 | 0.99728 | 0.99797 | 0.99856 |
|  | PC21 | PC22 | PC18 | PC19 | PC20 |
| Standard deviation | 0.001694 | 0.001498 | 0.002615 | 0.002341 | 0.002154 |
| Proportion of Variance | 0.00036 | 0.00029 | 0.00087 | 0.0007 | 0.00059 |
| Cumulative Proportion | 0.99893 | 0.99922 | 0.99728 | 0.99797 | 0.99856 |
|  | PC21 | PC22 | PC23 | PC24 | PC25 |
| Standard deviation | 0.001694 | 0.001498 | 0.001327 | 0.00112 | 0.0009947 |
| Proportion of Variance | 0.00036 | 0.00029 | 0.00022 | 0.00016 | 0.00013 |
| Cumulative Proportion | 0.99893 | 0.99922 | 0.99944 | 0.9996 | 0.99972 |
|  | PC26 | PC27 | PC28 | PC29 | PC30 |
| Standard deviation | 0.0006962 | 0.000681 | 0.0005303 | 0.0004486 | 0.0003342 |
| Proportion of Variance | 0.00006 | 0.00006 | 0.00004 | 0.00003 | 0.00001 |
| Cumulative Proportion | 0.99979 | 0.99984 | 0.99988 | 0.99991 | 0.99992 |
|  | PC31 |  |  |  |  |
| Standard deviation | 0.0003209 |  |  |  |  |
| Proportion of Variance | 0.00001 |  |  |  |  |
| Cumulative Proportion | 0.99993 |  |  |  |  |
