# Supplementary material for: Shared ecological traits influence shape of the skeleton in flatfishes (Pleuronectiformes)
Source: PeerJ. 2020 Apr 3;8:e8919. doi: 10.7717/peerj.8919 (PMC7134016; doi:10.7717/peerj.8919)
Supplement: Supplemental Information 3 — Significant ecological factors are highlighted in green. Significants codes as follows (***) 0; (**) 0.001; (*) 0.01; (.) 0.05. [file peerj-08-8919-s003.docx]

| **Procrustes ANOVA** | | | | | | | | |
| --- | --- | --- | --- | --- | --- | --- | --- | --- |
|  | Df | SS | MS | Rsq | F | Z | Pr(>F) |  |
| **depth** | 1 | 0.000268 | 0.00026802 | 0.01621 | 1.202 | 0.53166 | 0.298 |  |
| **climate** | 3 | 0.0011875 | 0.00039582 | 0.07181 | 1.7751 | 1.61143 | **0.053** | . |
| **water** | 2 | 0.0016604 | 0.0008302 | 0.10041 | 3.7231 | 2.69363 | **0.003** | ** |
| **food** | 5 | 0.0031702 | 0.00063403 | 0.19171 | 2.8434 | 3.0055 | **0.003** | ** |
| **sediment** | 4 | 0.0017988 | 0.00044969 | 0.10878 | 2.0167 | 2.06209 | **0.023** | * |
| **Residuals** | 32 | 0.0071354 | 0.00022298 | 0.4315 |  |  |  |  |
| **Total** | 47 | 0.0165365 |  |  |  |  |  |  |
