# Supplementary material for: Shared ecological traits influence shape of the skeleton in flatfishes (Pleuronectiformes)
Source: PeerJ. 2020 Apr 3;8:e8919. doi: 10.7717/peerj.8919 (PMC7134016; doi:10.7717/peerj.8919)
Supplement: Supplemental Information 4 — Ecological group comparisons that are significant are highlighted in green. Significants codes as follows (***) 0; (**) 0.001; (*) 0.01; (.) 0.05. [file peerj-08-8919-s004.docx]

| **Pairwise distances between means, plus statistics** | | | | | |
| --- | --- | --- | --- | --- | --- |
| **Depth Zone** | **d** | **UCL (95%)** | **Z** | **Pr > d** |  |
| bathyal:sublittoral | 0.04238666 | 0.06021723 | -0.1385355 | 0.547 |  |
|  |  |  |  |  |  |
| **Climate** | **d** | **UCL (95%)** | **Z** | **Pr > d** |  |
| Polar:Subtropical | 0.05317156 | 0.08390792 | -0.9241059 | 0.84 |  |
| Polar:Temperate | 0.04231132 | 0.07678968 | -0.8424524 | 0.796 |  |
| Polar:Tropical | 0.06979413 | 0.09687377 | -0.4595699 | 0.668 |  |
| Subtropical:Temperate | 0.03401604 | 0.06152212 | -0.7618033 | 0.764 |  |
| Subtropical:Tropical | 0.04571395 | 0.06441839 | 0.04067451 | 0.454 |  |
| Temperate:Tropical | 0.06939849 | 0.09397144 | 0.07688992 | 0.447 |  |
|  |  |  |  |  |  |
| **Water Type** | **d** | **UCL (95%)** | **Z** | **Pr > d** |  |
| marine:marine.brack | 0.05662336 | 0.07538307 | -0.0472451 | 0.49 |  |
| marine:marine.brack.fresh | 0.04984447 | 0.08754643 | -0.374118 | 0.603 |  |
| marine.brack:marine.brack.fresh | 0.0426128 | 0.07350541 | -0.8517913 | 0.81 |  |
|  |  |  |  |  |  |
| **Food Items** | **d** | **UCL (95%)** | **Z** | **Pr > d** |  |
| crust:crust.fish | 0.06103329 | 0.11690057 | -0.8241892 | 0.766 |  |
| crust:crust.poly | 0.03851084 | 0.09754034 | -1.3334733 | 0.937 |  |
| crust:crust.poly.ech.fish | 0.05341265 | 0.10754557 | -1.1843462 | 0.889 |  |
| crust:crust.poly.fish | 0.0490699 | 0.10271075 | -1.1330735 | 0.883 |  |
| crust:fish | 0.0608324 | 0.12668198 | -0.9054575 | 0.82 |  |
| crust.fish:crust.poly | 0.06376353 | 0.08286007 | -0.1322429 | 0.533 |  |
| crust.fish:crust.poly.ech.fish | 0.05896344 | 0.08794935 | -0.0766664 | 0.494 |  |
| crust.fish:crust.poly.fish | 0.02554881 | 0.05161446 | -1.0151403 | 0.849 |  |
| crust.fish:fish | 0.02120532 | 0.05458591 | -1.5112648 | 0.974 |  |
| crust.poly:crust.poly.ech.fish | 0.04470085 | 0.06487329 | -1.1166497 | 0.867 |  |
| crust.poly:crust.poly.fish | 0.04481226 | 0.05947744 | -0.0985134 | 0.502 |  |
| crust.poly:fish | 0.05673676 | 0.09493554 | -0.3770846 | 0.616 |  |
| crust.poly.ech.fish:crust.poly.fish | 0.03907708 | 0.05613906 | 0.1832972 | 0.405 |  |
| crust.poly.ech.fish:fish | 0.06465182 | 0.10463576 | -0.256266 | 0.552 |  |
| crust.poly.fish:fish | 0.02986984 | 0.07002015 | -1.1394459 | 0.907 |  |
|  |  |  |  |  |  |
| **Sediment** | **d** | **UCL (95%)** | **Z** | **Pr > d** |  |
| mud:mud.sand | 0.04476578 | 0.05416609 | 0.8404614 | 0.204 |  |
| mud:mud.sand.rock | 0.08743568 | 0.05835466 | 4.1925359 | 0.003 | ** |
| mud:sand | 0.01891573 | 0.03638695 | -0.3803596 | 0.592 |  |
| mud:sand.rock | 0.05427525 | 0.07302245 | 0.5311615 | 0.293 |  |
| mud.sand:mud.sand.rock | 0.04550526 | 0.06011189 | 0.6473375 | 0.238 |  |
| mud.sand:sand | 0.03845265 | 0.04526178 | 0.9888382 | 0.166 |  |
| mud.sand:sand.rock | 0.01977357 | 0.05077528 | -1.3341565 | 0.938 |  |
| mud.sand.rock:sand | 0.07835149 | 0.05476439 | 3.8521847 | 0.005 | ** |
| mud.sand.rock:sand.rock | 0.0414558 | 0.07713539 | -0.5136685 | 0.66 |  |
| sand:sand.rock | 0.04689528 | 0.06090409 | 0.652112 | 0.239 |  |
