# Supplementary figures and images for: Shared ecological traits influence shape of the skeleton in flatfishes (Pleuronectiformes)
Source: PeerJ. 2020 Apr 3;8:e8919. doi: 10.7717/peerj.8919 (PMC7134016; doi:10.7717/peerj.8919)

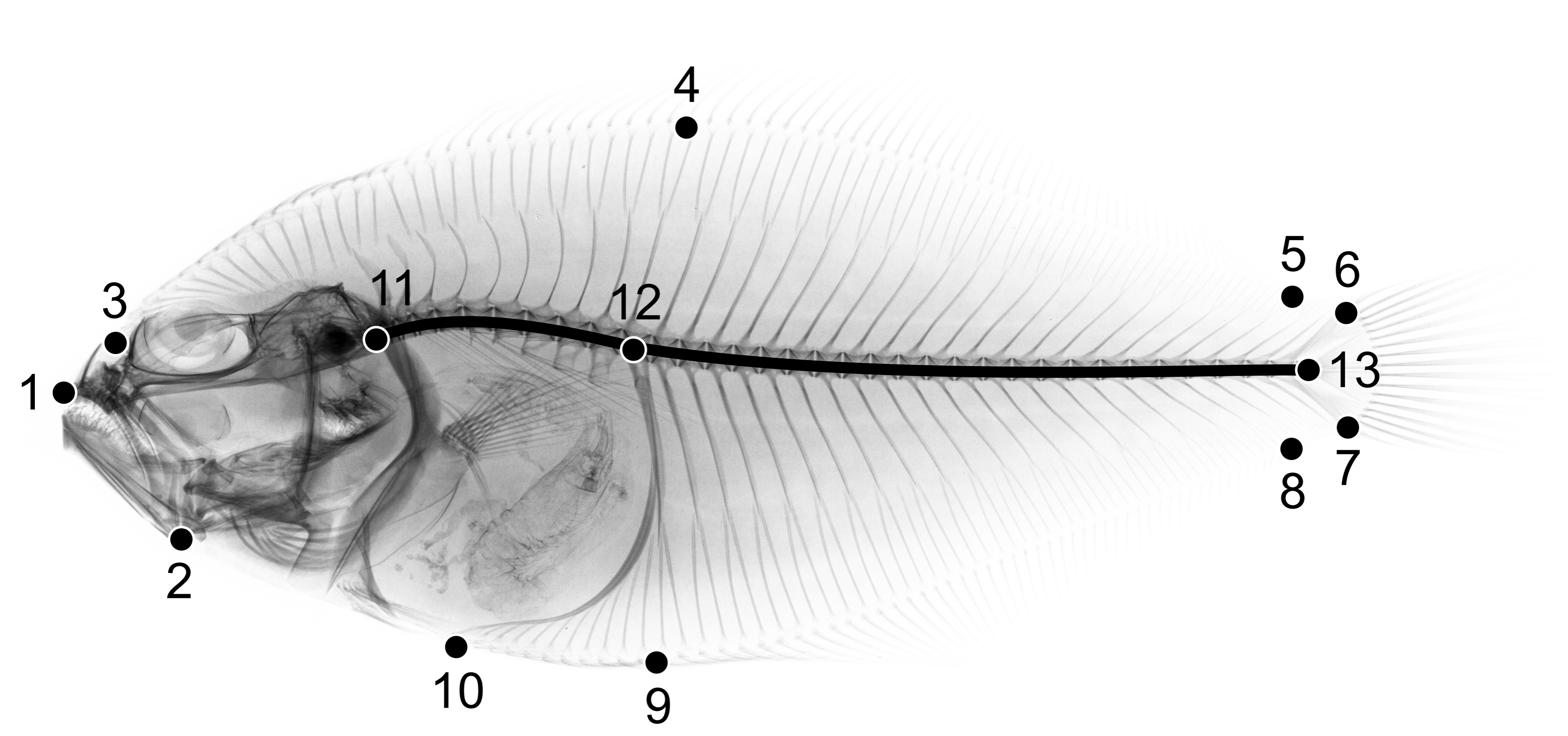

1

3

2

11

4

12

5

6

13

8

7

10

9

Supplement: Supplemental Information 5 — (1) anterior tip of premaxilla; (2) quadrate and articular junction; (3) origin of anterior dorsal fin; (4) dorsal basal bone between interneural spines of the first caudal vertebrae; (5) insertion of dorsal fin; (6) origin of caudal fin; (7) insertion of caudal fin; (8) insertion of anal fin; (9) ventral basal bone between interneural spines of the first caudal vertebrae; (10) origin of anal fin; (11) first abdominal vertebrae midpoint; (12) first caudal vertebrae midpoint; (13) urostyle midpoint. Curve containing 25 semi-landmarks following the spinal column. Syacium micrurum (Paralichthyidae). [file peerj-08-8919-s005.pdf]

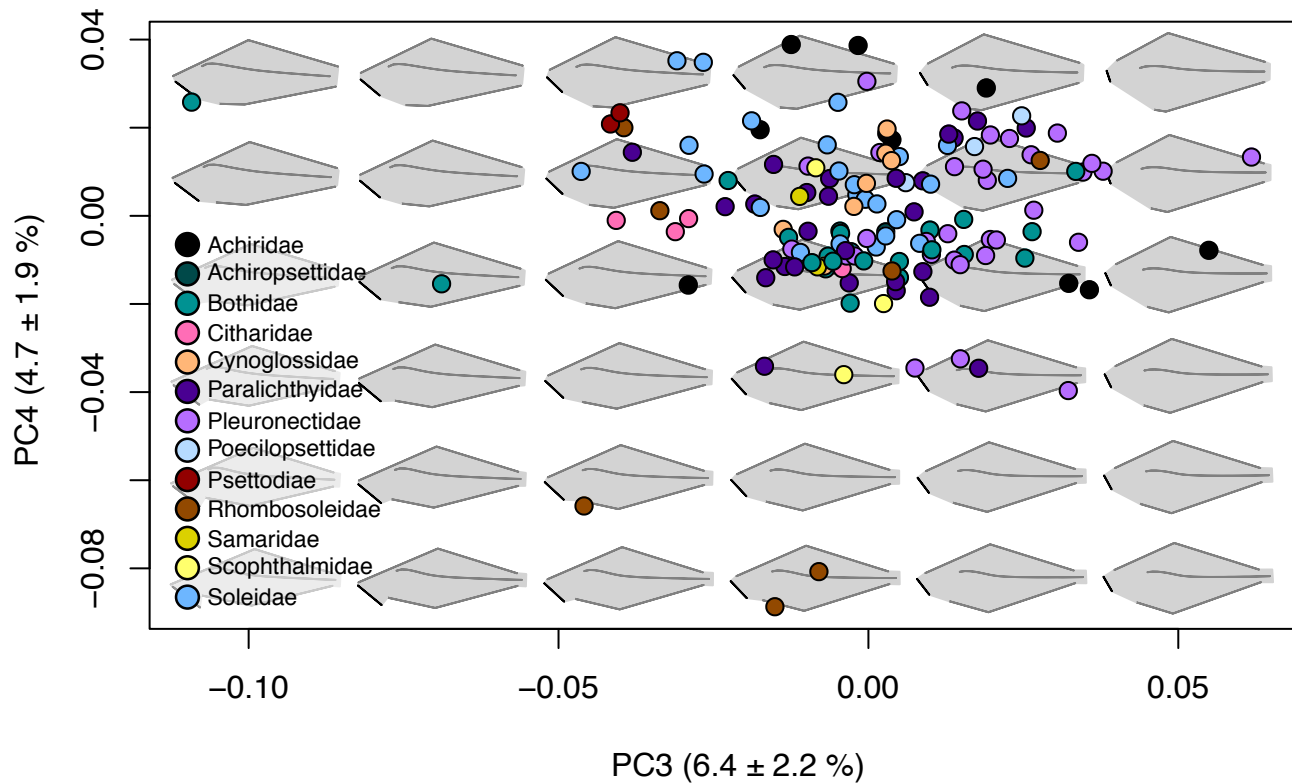

Supplement: Supplemental Information 7 — The morphospace biplot of PCs 3 and 4 represents the overall body shape variation within the flatfishes. Backtransform shapes (gray) portray shape variation throughout morphospace and fin length, jaw length and spinal curvature are shown on backtransform shapes as black lines. [file peerj-08-8919-s007.pdf]

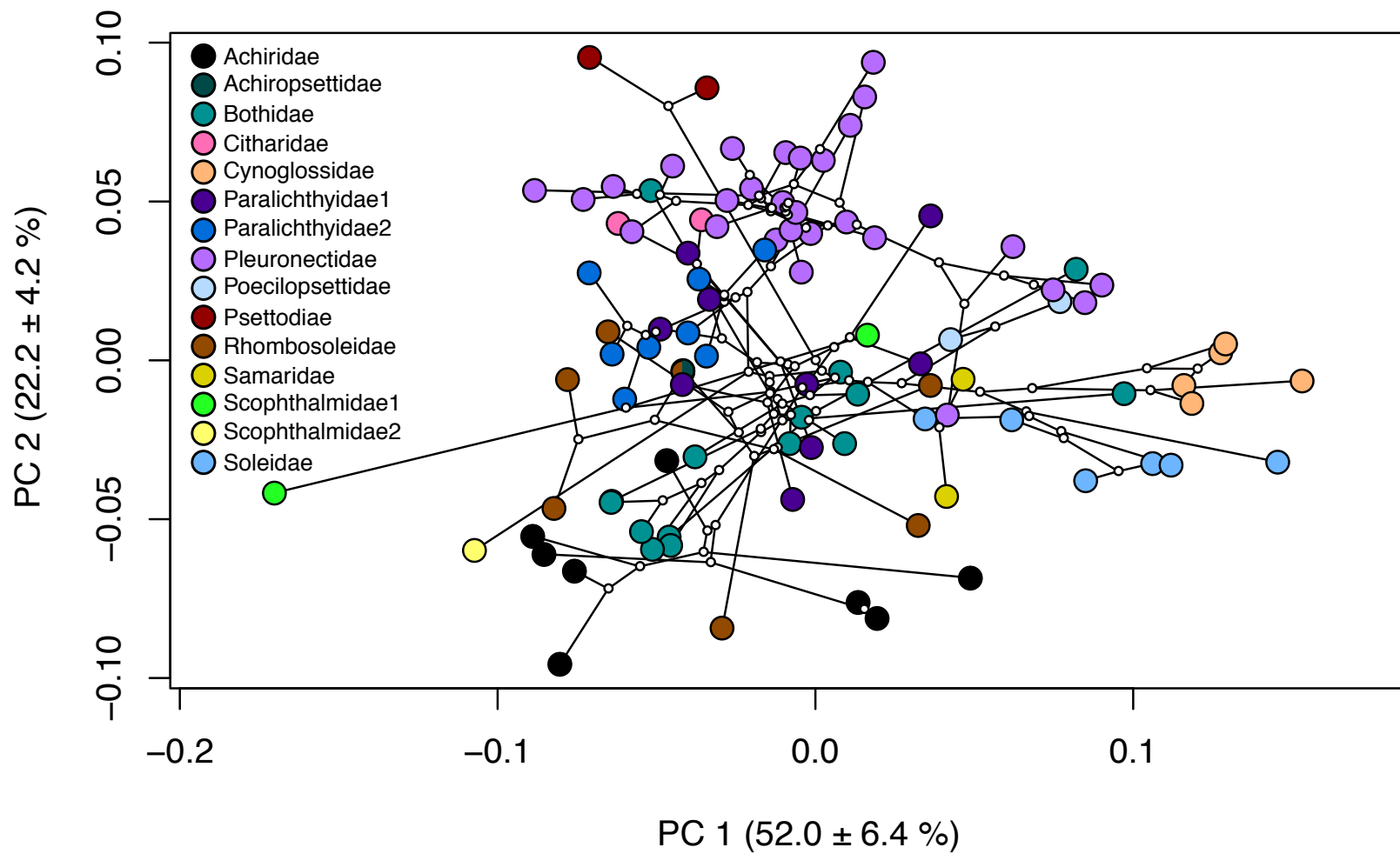

Supplement: Supplemental Information 8 — Phylomorphospace of the genomic phylogeny (Byrne, Chapleau & Aris-Brosou, 2018) for 98 species of flatfishes. Click on the colored circle next to the family name to circle the group. Show name of species by hovering over points on PCA. [file peerj-08-8919-s008.pdf]

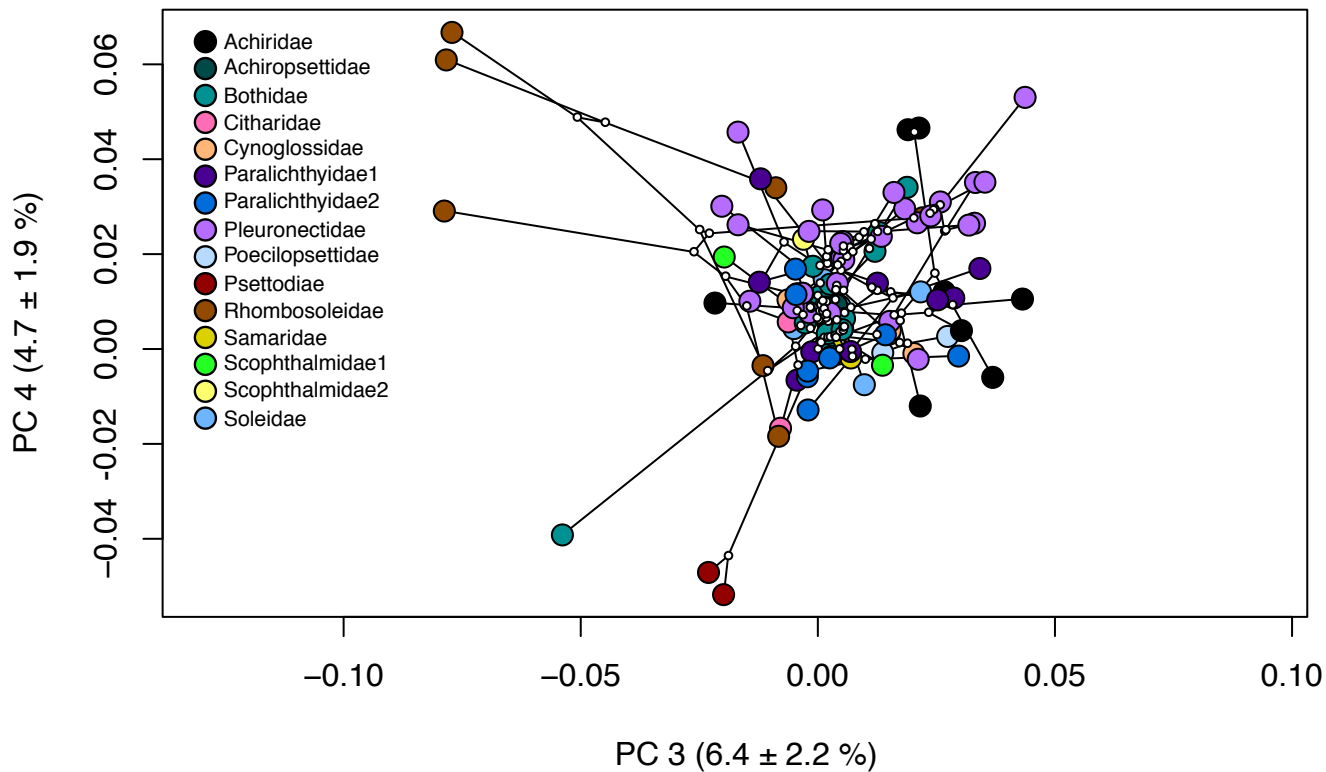

Supplement: Supplemental Information 9 — The genomic phylogeny (Byrne, Chapleau & Aris-Brosou, 2018) was mapped onto the morphospace biplot of PCs 1 and 2. Colors correlate to distinct clades. [file peerj-08-8919-s009.pdf]

**Observed K = 0.616 ; P-value = 0.001**

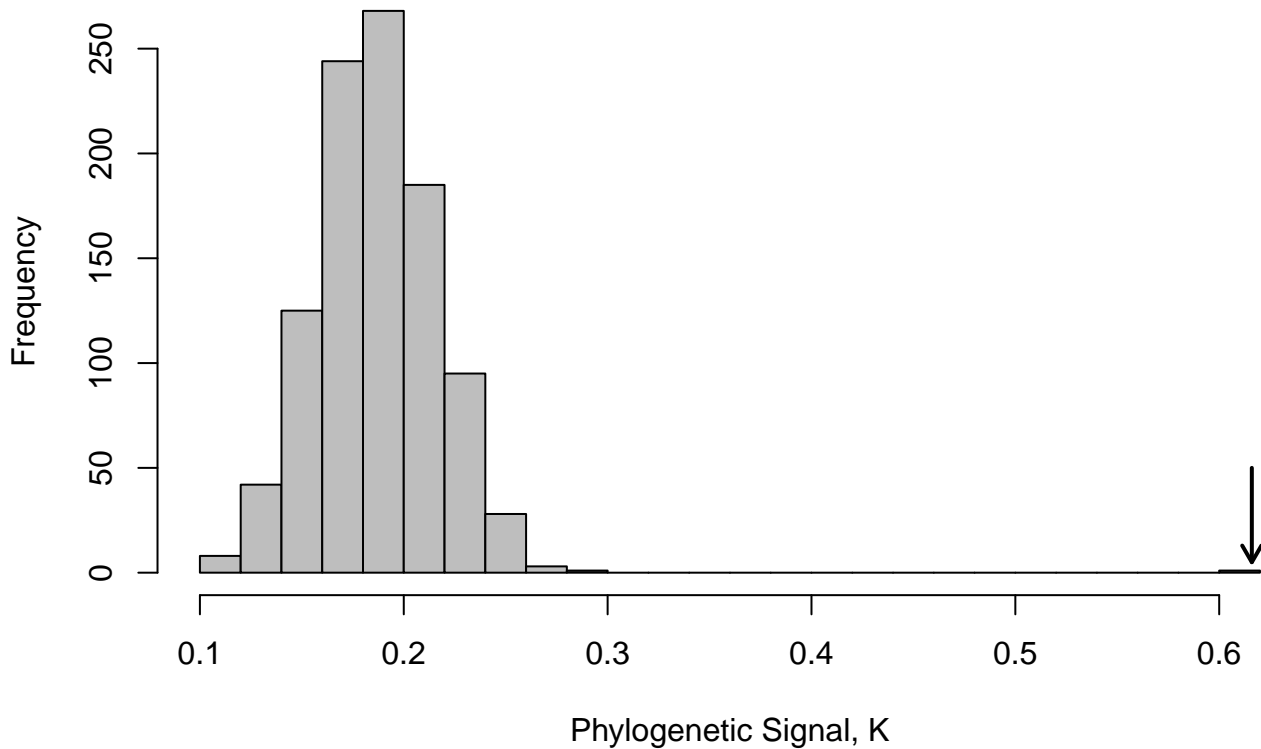

Supplement: Supplemental Information 12 — Phylogenetic signal is shown on x-axis and frequency on y-axis. Observed k value is noted along the x-axis by black arrow. [file peerj-08-8919-s012.pdf]

A

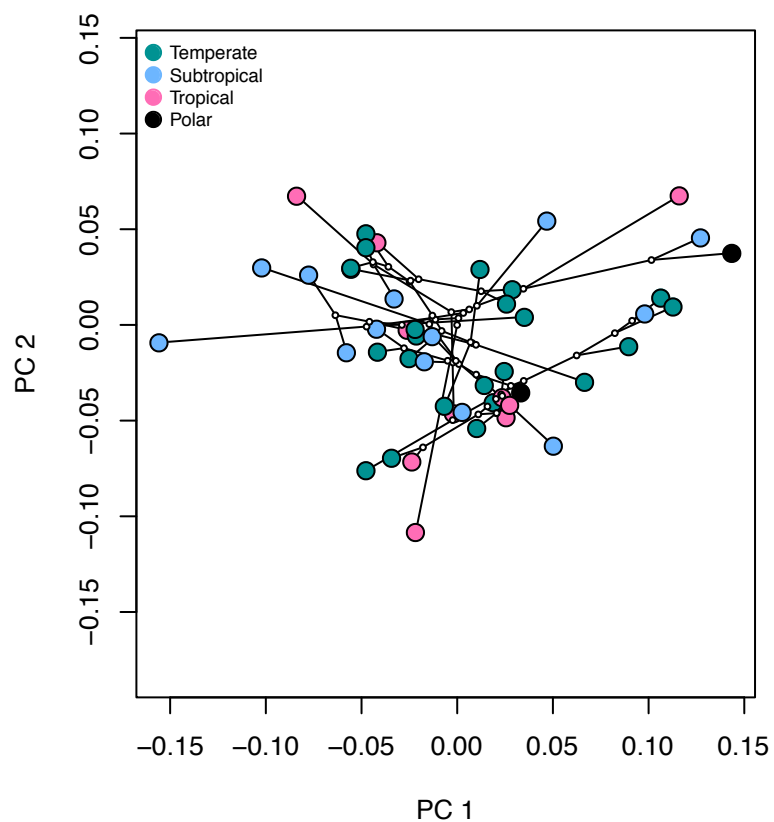

B

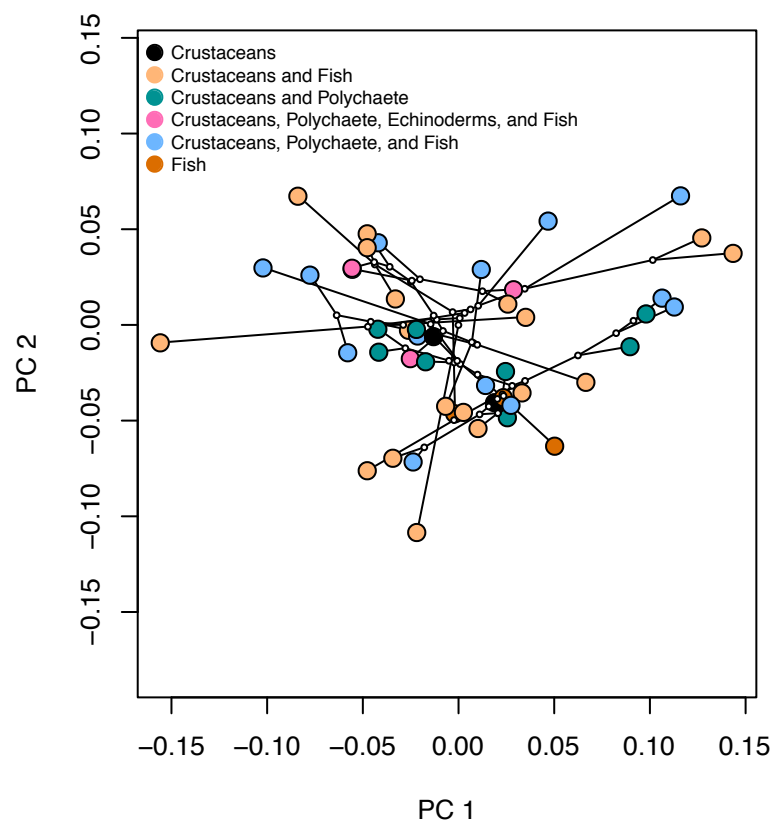

C

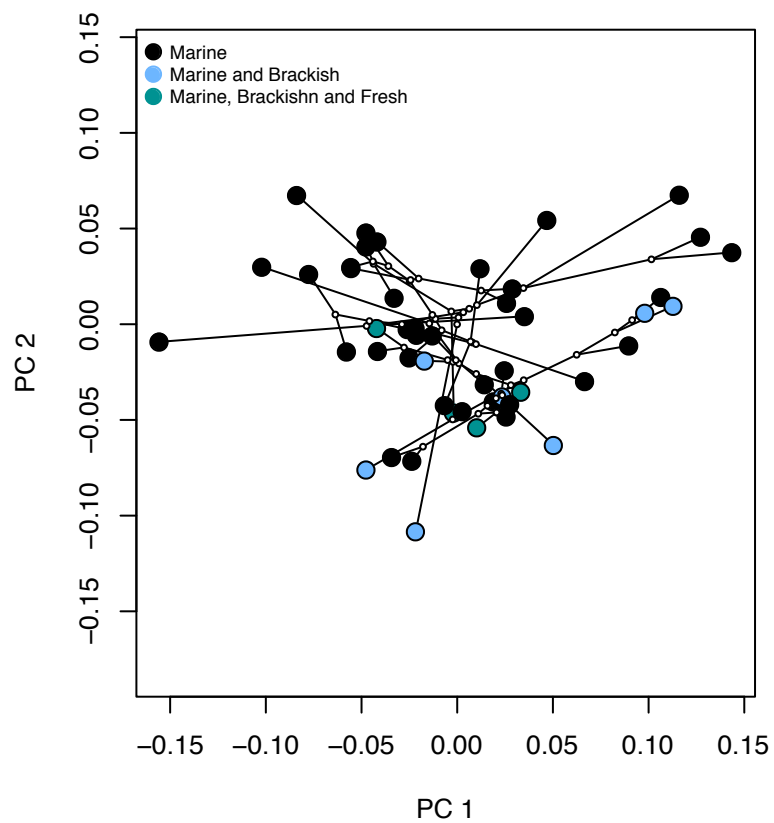

D

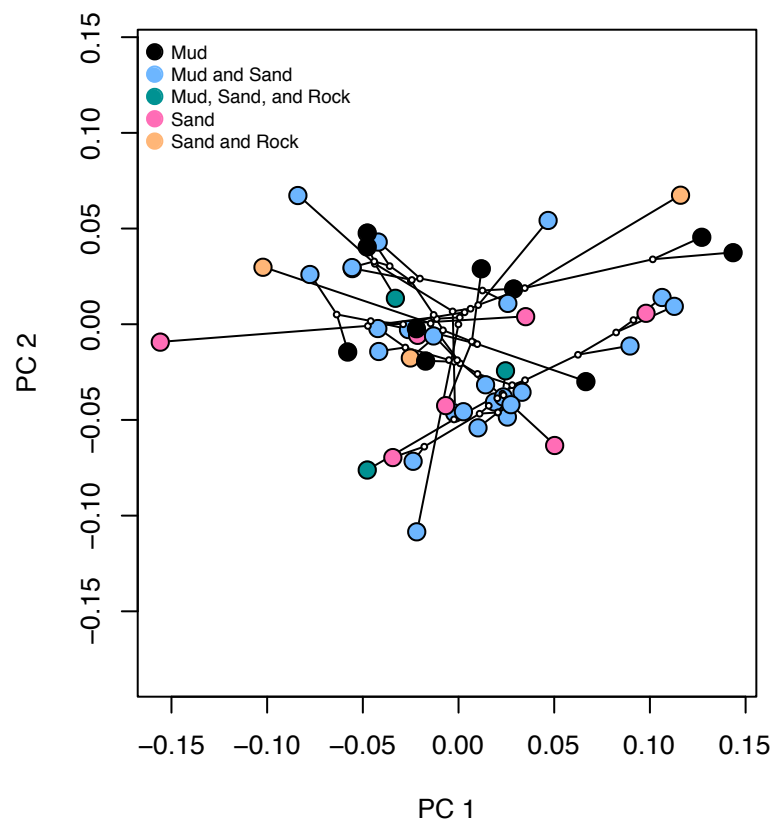

Supplement: Supplemental Information 13 — (A) climate type, (B) diet type, (C) water type and (D) sediment type. Colors correlate to distinct clades. [file peerj-08-8919-s013.pdf]
