# Supplementary material for: Shared ecological traits influence shape of the skeleton in flatfishes (Pleuronectiformes)
Source: PeerJ. 2020 Apr 3;8:e8919. doi: 10.7717/peerj.8919 (PMC7134016; doi:10.7717/peerj.8919)
Supplement: Supplemental Information 6 — Colors correlate to distinct clades in morphospace, phylomorphospace, and chronophylomorphospace. [file peerj-08-8919-s006.pdf]

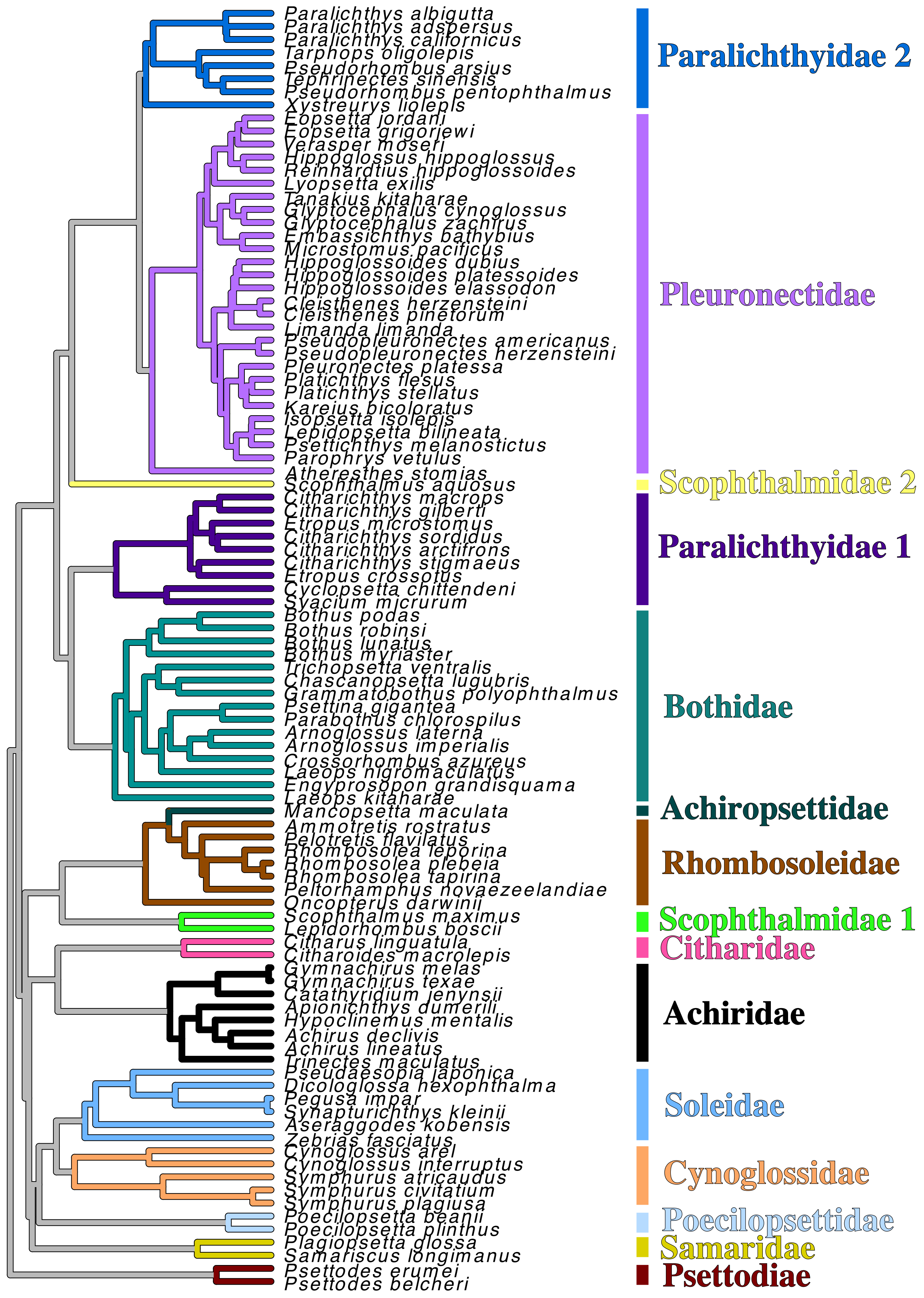
Paralichthys albigutta
Paralichthys adspersus
Paralichthys californicus
Tarphops oligolepis
Pseudorhombus arsius
Tephrinectes sinensis
Pseudorhombus pentophthalmus
Xystreurys liolepis
Eopsetta jordani
Eopsetta grigorjewi
Verasper moseri
Hippoglossus hippoglossus
Reinhardtius hippoglossoides
Lyopsetta exilis
Tanakius kitaharae
Glyptocephalus cynoglossus
Glyptocephalus zachirus
Embassichthys bathybius
Microstomus pacificus
Hippoglossoides dubius
Hippoglossoides platessoides
Hippoglossoides elassodon
Cleisthenes herzensteini
Cleisthenes pinetorum
Limanda limanda
Pseudopleuronectes americanus
Pseudopleuronectes herzensteini
Pleuronectes platessa
Platichthys flesus
Platichthys stellatus
Kareius bicoloratus
Isopsetta isolepis
Lepidopsetta bilineata
Psettichthys melanostictus
Parophrys vetulus
Atheresthes stomias
Scophthalmus aquosus
Citharichthys macrops
Citharichthys gilberti
Etropus microstomus
Citharichthys sordidus
Citharichthys arctifrons
Citharichthys stigmaeus
Etropus crossotus
Cyclopsetta chittendeni
Syacium micrurum
Bothus podas
Bothus robinsi
Bothus lunatus
Bothus myriaster
Trichopsetta ventralis
Chascanopsetta lugubris
Grammatobothus polyophthalmus
Psettina gigantea
Parabothus chlorospilus
Arnoglossus laterna
Arnoglossus imperialis
Crossorhombus azureus
Laeops nigromaculatus
Engyprosopon grandisquama
Laeops kitaharae
Mancopsetta maculata
Ammotretis rostratus
Pelotretis flavilatus
Rhombosolea leporina
Rhombosolea plebeia
Rhombosolea tapirina
Peltorhamphus novaezeelandiae
Oncopterus darwinii
Scophthalmus maximus
Lepidorhombus boscii
Citharus linguatula
Citharoides macrolepis
Gymnachirus melas
Gymnachirus texae
Catathyridium jenynsii
Apionichthys dumerili
Hypoclinemus mentalis
Achirus declivis
Achirus lineatus
Trinectes maculatus
Pseudaesopia japonica
Dicologlossa hexophthalma
Pegusa impar
Synapturichthys kleinii
Aseraggodes kobensis
Zebrias fasciatus
Cynoglossus arel
Cynoglossus interruptus
Symphurus atricaudus
Symphurus civitatium
Symphurus plagiusa
Poecilopsetta beanii
Poecilopsetta plinthus
Plagiopsetta glossa
Samariscus longimanus
Psettodes erumei
Psettodes belcheri
Paralichthyidae 2
Pleuronectidae
Scophthalmidae 2
Paralichthyidae 1
Bothidae
Achiropsettidae
Rhombosoleidae
Scophthalmidae 1
Citharidae
Achiridae
Soleidae
Cynoglossidae
Poecilopsettidae
Samaridae
Psettodiae
