# Supplementary material for: Shared ecological traits influence shape of the skeleton in flatfishes (Pleuronectiformes)
Source: PeerJ. 2020 Apr 3;8:e8919. doi: 10.7717/peerj.8919 (PMC7134016; doi:10.7717/peerj.8919)
Supplement: Supplemental Information 10 — The genomic phylogeny was mapped onto the three dimensional morphospace for PCs 1, 2 and 3. Colors correlate to distinct clades. [file peerj-08-8919-s010.zip › Supplemental Figure S6.html]

 
RGL model


You must enable Javascript to view this page properly.

  
Drag mouse to rotate model. Use mouse wheel or middle button
to zoom it.

---

  
Object written from rgl 0.100.30 by writeWebGL.
